# Benefit of Linked‐Color Imaging in Artificial Intelligence‐Assisted Diagnosis of Early Gastric Cancer: A Pilot Study With Propensity Score Adjustment

**DOI:** 10.1002/deo2.70381

**Published:** 2026-07-28

**Authors:** Ahmed Nashaat Mohamed, Yosuke Minoda, Ryohei Maruoka, Yusuke Suzuki, Hiroki Fukuya, Hirotaka Tsuru, Masafumi Wada, Yoshimasa Tanaka, Takatoshi Chinen, Tomohiko Moriyama, Haruei Ogino, Eikichi Ihara, Yoshihiro Ogawa

**Affiliations:** ^1^ Department of Medicine and Bioregulatory Science, Graduate School of Medical Sciences Kyushu University Fukuoka Japan; ^2^ International Medical Department Kyushu University Hospital Fukuoka Japan; ^3^ The National Hepatology and Tropical Medicine Research Institute Cairo Egypt; ^4^ Department of Endoscopic Diagnostics and Therapeutics Kyushu University Hospital Fukuoka Japan

**Keywords:** artificial intelligence, early gastric cancer, endoscopy, image‐enhanced endoscopy, linked‐color imaging

## Abstract

**Background and Aims:**

Artificial intelligence (AI)‐assisted endoscopy represents a promising approach for lesion detection, yet frequent false‐positive detections impair clinical utility by disrupting examinations and diminishing physician confidence. Linked‐color imaging (LCI), an image‐enhanced endoscopy technique that amplifies mucosal and vascular contrast, may address this limitation. This investigation evaluated whether LCI reduces false‐positive AI detections compared with white‐light imaging (WLI).

**Methods:**

This retrospective study analyzed consecutive AI‐assisted upper endoscopies performed between March 2024 and June 2025. WLI and LCI were performed sequentially within the same endoscopic session in each patient. False‐positive AI detections were compared between modalities using two computer‐aided detection (CAD) versions. Propensity score adjustment was used as a sensitivity analysis for baseline differences between CAD Versions I and II.

**Results:**

Of 66 initially screened cases, 63 remained after excluding patients with prior gastric surgery. LCI reduced false‐positive AI detections compared with WLI (median 2 vs. 5; *p* < 0.001). In CAD version–stratified sensitivity analyses, LCI reduced false‐positive AI detections in both Version I (5 to 2; *p* = 0.01) and Version II (2 to 0; *p* = 0.03). This reduction remained consistent across atrophic grades. Both imaging modalities identified all gastric lesions, achieving 100% detection sensitivity.

**Conclusions:**

LCI assessment performed after WLI observation yielded fewer false‐positive CAD‐EYE detections while maintaining lesion detection sensitivity. However, because the observation sequence was fixed, these findings should be interpreted cautiously and require confirmation in prospective or counterbalanced studies.

**Trial Registration**: N/A (retrospective study).

## 1 Introduction

Gastric cancer persists as a major cause of cancer mortality in East Asia, with Japan reporting over 110,000 new cases annually despite advances in screening and therapeutic endoscopy [1]. National screening programs have enhanced early detection rates, yet identifying subtle early‐stage lesions remains challenging for endoscopists [2, 3].

Computer‐aided detection systems utilizing artificial intelligence (AI) have emerged to augment lesion identification [3, 4, 5]. However, clinical implementation faces obstacles including limited clinical evidence, frequent false‐positive detections disrupting examinations, and uncertainty regarding strategies to minimize such inaccuracies [6, 7, 8, 9].

Image‐enhanced endoscopy demonstrates improved diagnostic performance for gastric neoplastic and preneoplastic conditions [9, 10]. Linked‐color imaging (LCI), among available image‐enhanced modalities, exhibits particular value in accentuating color differences in early gastric cancer and enhancing lesion conspicuity [11, 12, 13, 14, 15, 16]. While image‐enhanced endoscopy benefits human observers, its spectral properties may also affect AI interpretation of mucosal patterns, a relationship requiring investigation [17, 18]. The enhanced color contrast and textural definition provided by LCI may facilitate superior AI discrimination of lesion boundaries relative to white‐light imaging (WLI) [4, 12, 17].

This investigation directly compares LCI with WLI in AI‐assisted examinations. The primary objective was to determine whether LCI could reduce the elevated false‐positive detection rates associated with AI‐assisted endoscopy, thereby improving clinical utility [4].

## 2 Methods

### 2.1 Study Design and Patient Selection

**FIGURE 1:**
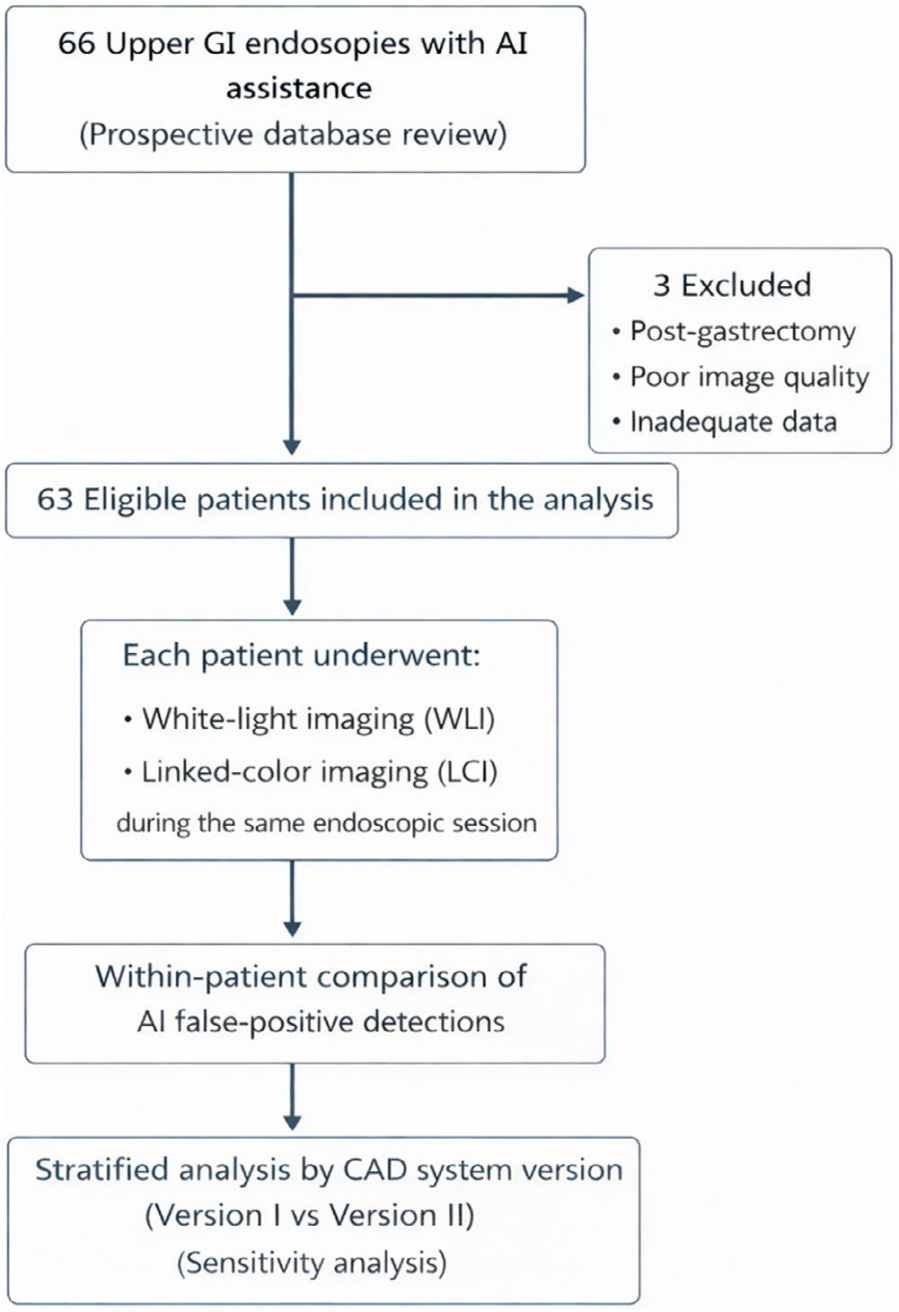
Flowchart of patient inclusion and study design. Of 66 upper gastrointestinal endoscopies performed with artificial intelligence (AI) assistance, three cases were excluded because of post‐gastrectomy status, poor image quality, or inadequate data. The remaining 63 patients were included in the analysis. In each patient, white‐light imaging (WLI) and linked‐color imaging (LCI) were performed sequentially during the same endoscopic session. False‐positive AI detections were compared using a within‐patient design, with additional stratified analyses according to CAD system version.

This retrospective observational investigation utilized data from adult patients undergoing upper gastrointestinal endoscopy at Kyushu University Hospital between March 2024 and June 2025. Initial screening identified 66 cases examined using computer‐aided detection systems. Three cases with prior gastric surgery were excluded, yielding 63 cases for analysis (Figure 1). The cohort included six patients with known gastric tumors before endoscopy. WLI and LCI were performed sequentially within the same patient during a single endoscopic session using real‐time modality switching. Procedures employed either Version I or Version II of the FUJIFILM computer‐aided detection AI system for LCI and WLI comparison [13]. Demographic and clinical indication data were collected (Table 1). The institutional review board approved this study with waived individual informed consent, given the retrospective design; the protocol was publicly announced via an opt‐out mechanism.

**TABLE 1:** Baseline characteristics of the eligible patients according to computer‐aided detection (CAD) version.

Characteristic	Overall cohort (*n* = 63)	CAD Version I (*n* = 39)	CAD Version II (*n* = 24)	SMD
Age, median (IQR), years	64 (52.5–72.5)	63.5 (52.8–73.5)	62.0 (51.3–73.8)	0.38
Male sex, *n* (%)	32 (50.8)	18 (46.2)	14 (58.3)	0.24
Female sex, *n* (%)	31 (49.2)	21 (53.8)	10 (41.7)	0.24
Indication				
Screening, *n* (%)	32 (50.8)	21 (53.8)	11 (45.8)	0.16
Surveillance, *n* (%)	29 (46.0)	18 (46.2)	11 (45.8)	0.01
Follow‐up after EMR, *n* (%)	2 (3.2)	0 (0.0)	2 (8.3)	0.43
Gastric mucosal atrophy (Kimura–Takemoto)				
Closed type, *n* (%)	50 (79.4)	30 (76.9)	20 (83.3)	0.16
Open type, *n* (%)	13 (20.6)	9 (23.1)	4 (16.7)	0.16
CAD Version				
Version I, *n* (%)	39 (61.9)	39 (100)	0 (0.0)	—
Version II, *n* (%)	24 (38.1)	0 (0.0)	24 (100)	—

*Note*: Table 1 summarizes the 63 eligible patients after exclusion of three patients from the initially screened cohort of 66 patients. CAD version rows are descriptive by definition and were not used for SMD interpretation. Abbreviations: CAD, computer‐aided detection; EMR, endoscopic mucosal resection; IQR, interquartile range; SMD, standardized mean difference.

The AI‐generated detection results were recorded after each endoscopic examination as part of routine system performance monitoring. These results were subsequently reviewed independently by three experienced endoscopists, who classified each AI‐flagged lesion as a true‐positive or false‐positive finding based on endoscopic and, when available, histologic assessment.

### 2.2 AI System and Endoscopy Protocol

#### 2.2.1 AI System Usage during Endoscopic Procedures

The FUJIFILM computer‐aided detection AI system (CAD system) employs deep learning algorithms to automatically identify suspicious lesions in real‐time during both WLI and LCI examinations [2, 4]. In this study, AI‐assisted detection was performed during all procedures, rather than being retrospectively applied to stored images. Seven experienced gastroenterology specialists performed all procedures. Biopsy decisions reflected endoscopist clinical judgment supplemented by AI notifications. Expert gastrointestinal pathologists reviewed all pathological specimens.

Two versions of the CAD system were used during the study period. CAD Version I represents the initial iteration of the algorithm and was primarily optimized to achieve high sensitivity for gastric lesion detection. CAD Version II is a subsequent refined version trained on a larger and more diverse dataset, including common non‐neoplastic mimics, with the aim of improving specificity and reducing false‐positive detections [3].

#### 2.2.2 Endoscopic Imaging Protocol

All AI detections and adjudications in this study were based on non‐magnified high‐definition endoscopic views obtained during standard screening examinations. As endoscopic imaging modalities, WLI and LCI were performed sequentially during a single endoscopic session using real‐time modality switching. In all included examinations, WLI observation was performed first, followed by LCI observation according to a standardized imaging sequence. AI outputs generated under each imaging modality were analyzed separately. Blue light imaging was not included in the analysis, as AI‐assisted gastric lesion detection using the CAD system evaluated in this study is limited to WLI and LCI.

### 2.3 The Adjudication and Biopsy Protocol

A total of 136 AI‐flagged lesions from 63 cases were independently re‐evaluated by three expert endoscopists. Biopsy decisions for AI‐flagged lesions were made selectively at the discretion of the endoscopist based on real‐time visual assessment following an AI alert.　Based on endoscopic and histologic findings, the lesions were classified into three categories: Category (1) included six boxes corresponding to known neoplastic lesions, all of which had been previously biopsied and histologically confirmed at referring institutions. Category (2) included 21 AI‐flagged lesions that were classified as indeterminate on expert review. Among these lesions, those that had undergone targeted biopsy at the discretion of the endoscopist were adjudicated based on histologic findings. Category (3) included 109 lesions classified as definite non‐neoplasia. These lesions generally did not undergo biopsy and were adjudicated based on an independent review followed by a consensus assessment by three expert endoscopists, who classified the lesions as non‐neoplastic based on characteristic benign endoscopic features. One lesion in this category had undergone biopsy; however, histology confirmed non‐neoplastic pathology, and the lesion was ultimately adjudicated as non‐neoplastic.

### 2.4 Instruments and Endoscopists

All procedures were performed using the Fujifilm ELUXEO EP‐7000 system in combination with Fujifilm upper gastrointestinal endoscopes (EG‐840N, EG‐840T, EG‐840TP, and EG‐L600WR7). Seven participating endoscopists made biopsy decisions based on clinical judgment, utilizing AI output as supporting information.

### 2.5 Primary and Secondary Endpoints

The primary endpoint was the number of false‐positive AI detections per examination. Secondary endpoints included the total number of AI‐detections per examination, lesion detection sensitivity, biopsy frequency, and histologically confirmed neoplasms [8].

### 2.6 Definition of False Positives

False‐positive AI detection was defined as AI‐flagged lesions judged non‐neoplastic by endoscopic assessment or histological evaluation when available, as described in the “Adjudication and Biopsy Protocol” section [19]. This definition encompasses non‐neoplastic or artifactual detections (mucosal folds, bubbles, and benign polyps) rather than diagnostic misclassification [8].

### 2.7 Statistical Analysis

All statistical analyses were performed using R statistical software version 4.2.1 (R Foundation for Statistical Computing, Vienna, Austria). Continuous variables are presented as medians with interquartile ranges (IQRs). Within‐patient comparisons between WLI and LCI were performed using paired statistical analyses. Stratified analyses were conducted according to CAD version. Propensity score matching was applied only as a supplementary sensitivity analysis. Statistical significance was defined as *p* < 0.05.

### 2.8 Propensity Score Adjustment for Sensitivity Analysis

A propensity score–based adjustment was performed as a supplementary sensitivity analysis to assess the robustness of version‐level comparisons, because CAD Version I and Version II were used in independent patient cohorts. Consistent with this study design, propensity score matching was used as a sensitivity analysis rather than as a definitive approach to control for selection bias [20]. Propensity scores for CAD versions assignment were estimated using a multivariable logistic regression model including age, sex, clinical indication for endoscopy, and gastric mucosal atrophy status. Clinical indication was categorized as screening, surveillance, or follow‐up after endoscopic mucosal resection. Gastric mucosal atrophy status was classified as closed type or open type according to the Kimura–Takemoto classification. One‐to‐one nearest neighbor matching without replacement was performed with a 0.2 standard deviation caliper width of the propensity score logit. Standardized mean differences assessed covariate balance, with values below 0.1 indicating adequate equilibrium. Post‐matching diagnostics confirmed group comparability. All propensity score model variables had complete data without requiring imputation. Covariate balance before and after matching is presented in Table S1A.

## 3 Results

### 3.1 Baseline Characteristics

Baseline characteristics of the study population are summarized in Table 1. The median age of the study population was 64 years (IQR, 52.5–72.5). According to the Kimura–Takemoto classification, gastric mucosal atrophy was classified as closed type in 50 patients and open type in 13 patients. The detailed distribution of Kimura–Takemoto atrophy grades is presented in Table S1B. During the study period, 39 patients were examined using CAD Version I and 24 using CAD Version II. Baseline patient characteristics according to CAD version are summarized in Table 1. Median procedure times were 10.5 min (IQR, 6.0–14.0) with WLI and 11.0 min (IQR, 7.0–15.5) with LCI.

### 3.2 Primary Outcome: LCI Reduces False‐Positive AI Detection

**FIGURE 2:**
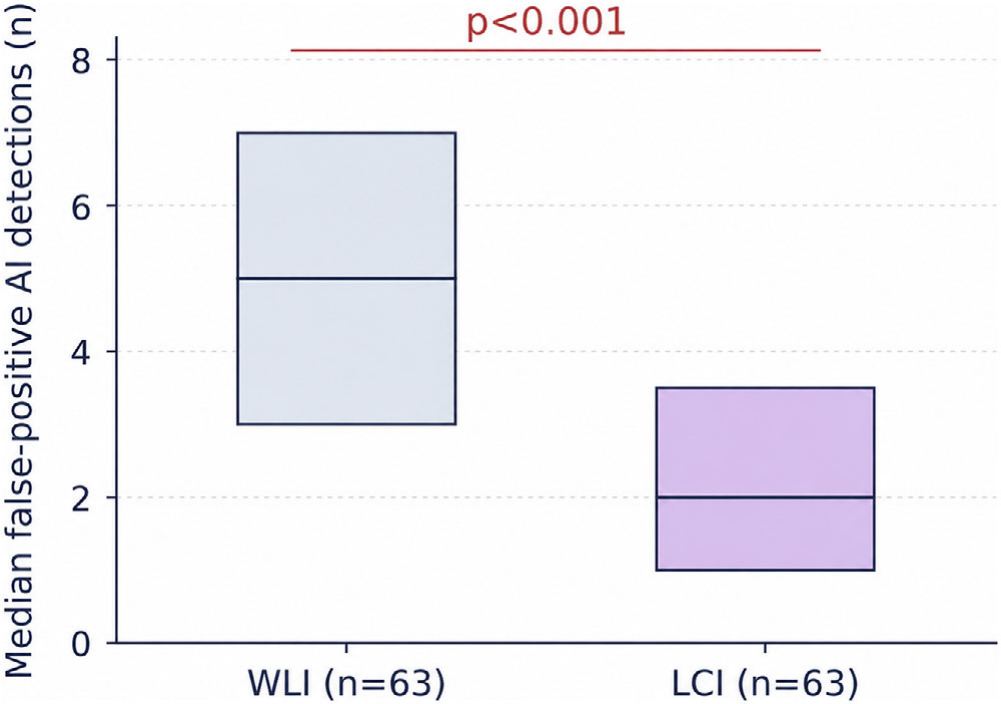
Overall within‐patient comparison of false‐positive artificial intelligence (AI) detections between WLI and LCI. LCI significantly reduced the number of false‐positive AI detections per examination compared with WLI in the overall cohort of 63 patients. The box indicates the interquartile range, and the central line indicates the median. WLI, white‐light imaging; LCI, linked‐color imaging. *p < 0.001.

In within‐patient comparisons, LCI was associated with a significantly lower median number of false‐positive AI detections per examination compared with WLI (median 2 vs. 5, *p* < 0.001) (Figure 2). This improvement in specificity did not compromise sensitivity, as the AI system maintained a 100% detection rate for all known and incident gastric tumors with both imaging modalities.

### 3.3 Secondary Outcome: Biopsy Outcomes and Detection Performance

**FIGURE 3:**
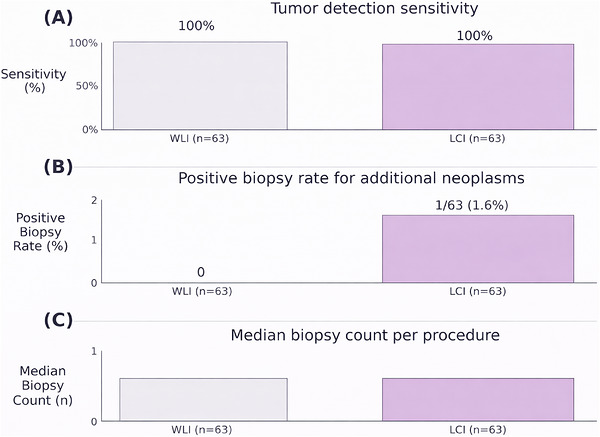
Biopsy Outcomes, tumor detection sensitivity, and additional lesion detection. Bar graphs compare diagnostic outcomes between linked‐color imaging (LCI) and white‐light imaging (WLI). between the linked‐color imaging and white‐light imaging groups. (A) Tumor detection sensitivity. (B) Positive biopsy rate for additional neoplasms. (C) Median biopsy count per procedure. Values are presented as percentages or counts per procedure as indicated. Gray boxes represent white‐light imaging, and purple boxes represent linked‐color imaging.

Median biopsy count per procedure was 0.0 (IQR 0–1.0) without difference between WLI and LCI examinations (*p* = 0.82). One adenoma was additionally detected via biopsy in the LCI examination (1/63), yielding a 1.6% positive biopsy rate for that cohort. The WLI group had no additional proven neoplasms (Figure 3).

### 3.4 Sub‐Analysis: Impact of CAD Version, Anatomical Location, and Gastric Atrophy

**FIGURE 4:**
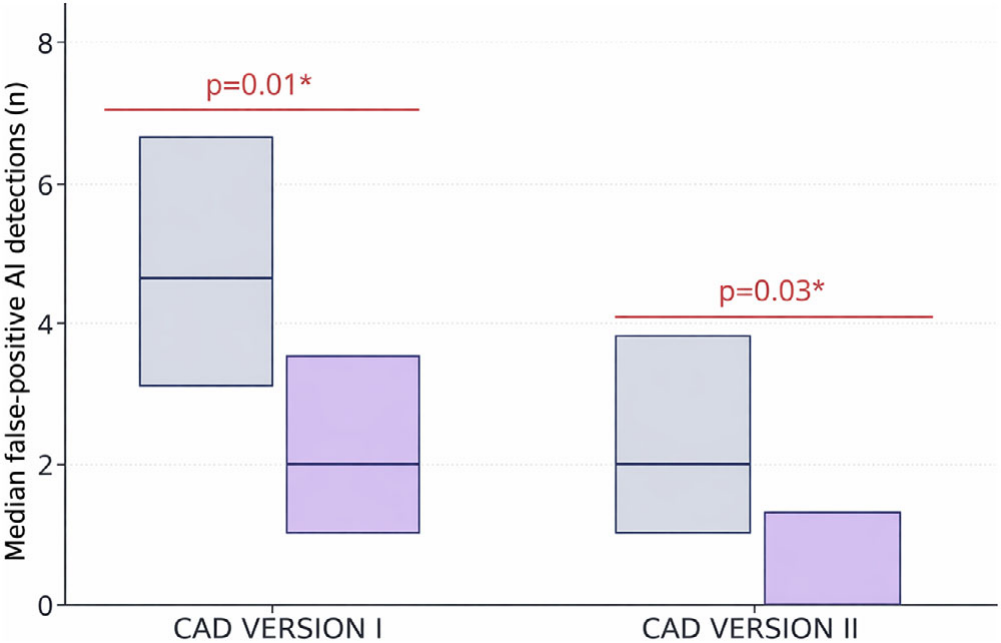
Comparison of false‐positive artificial intelligence (AI) detections between white‐light imaging and linked‐color imaging. Box plots display median false‐positive AI detection counts under white‐light imaging and linked‐color imaging for both computer‐aided detection system versions. Boxes represent medians with interquartile ranges. *p < 0.05 indicates statistical significance. Gray boxes represent white‐light imaging, and purple boxes represent linked‐color imaging.

LCI benefit was particularly evident in challenging clinical scenarios. Analyses stratified by CAD version were performed to further characterize AI performance. In Version I examinations, the median number of false‐positive AI detections decreased from 5 (IQR 3–7) with WLI to 2 (IQR 1–3.5) with LCI (*p* = 0.01). In Version II examinations, the median decreased from 2 (IQR 1–4) with WLI to 0 (IQR 0–1) with LCI (*p* = 0.03). Under the lowest CAD threshold setting, the lowest false‐positive detection was observed with LCI combined with CAD Version II, with a median of 0 false‐positive detections per examination (IQR 0–1) (Figure 4).

**FIGURE 5:**
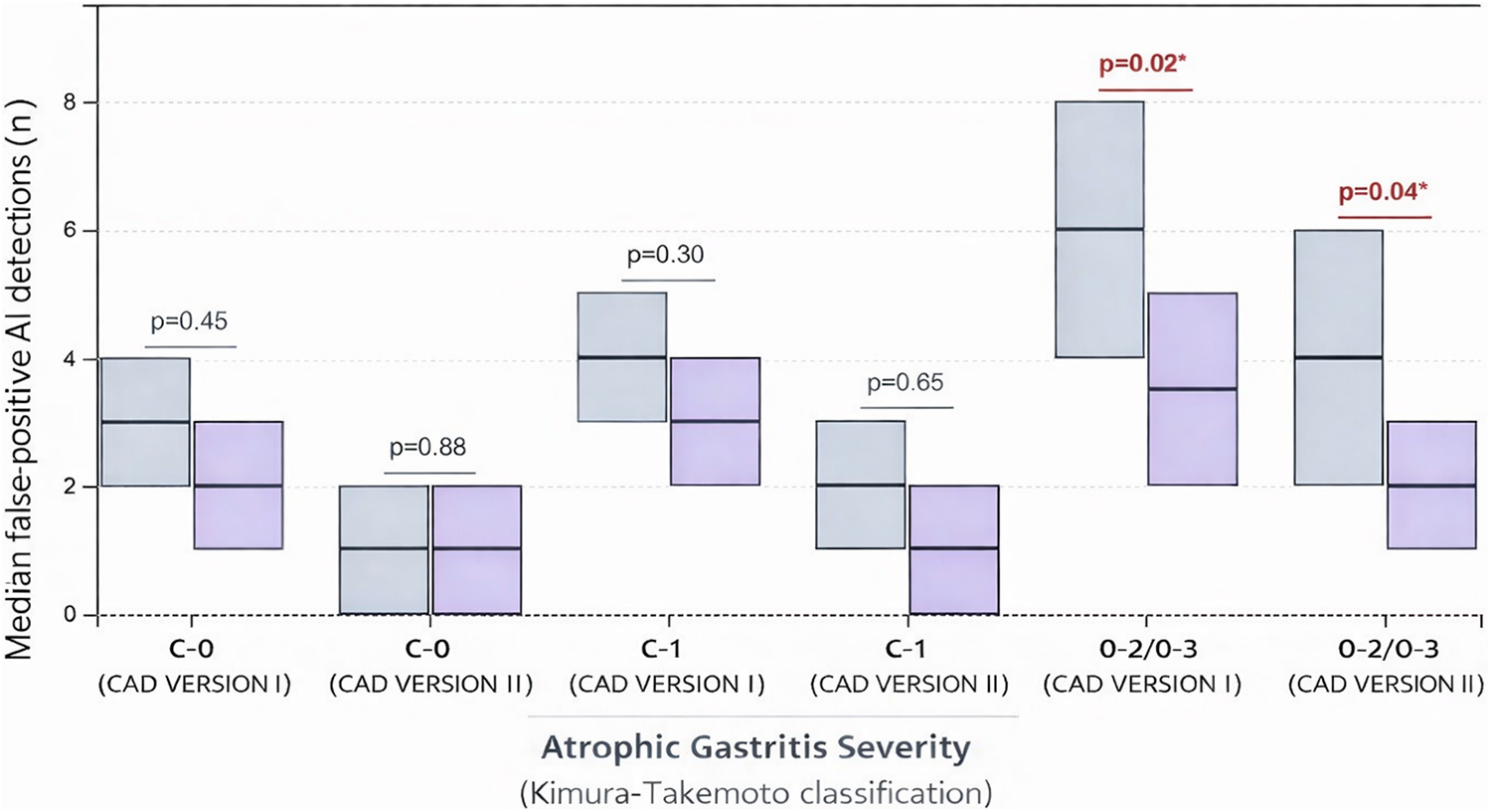
Effect of linked‐color imaging on false‐positive detections across atrophic gastritis severity. Box plots illustrate false‐positive AI detection counts stratified by gastric mucosal atrophy grade (Kimura‐Takemoto classification), comparing linked‐color imaging with white‐light imaging. Boxes represent medians with interquartile ranges. *p < 0.05 indicates statistical significance. Gray boxes represent white‐light imaging, and purple boxes represent linked‐color imaging.

**FIGURE 6:**
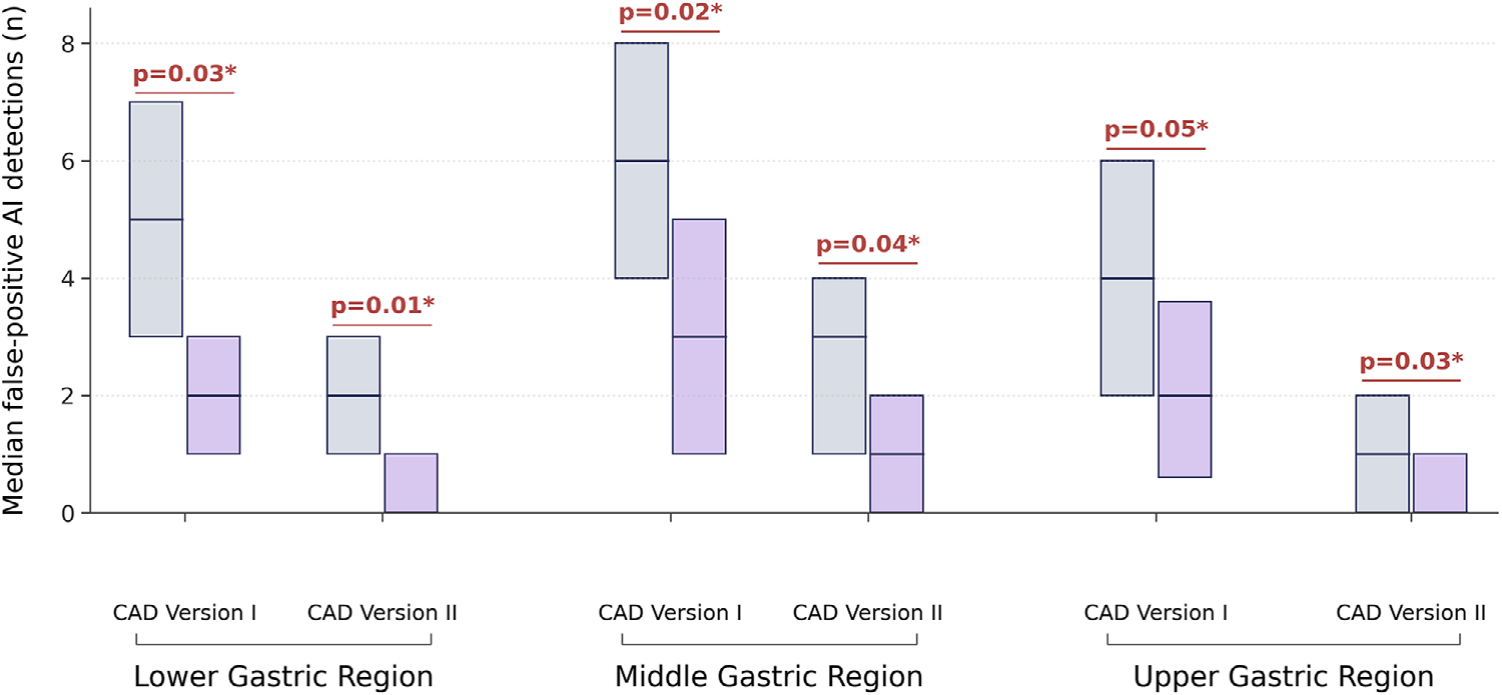
False‐positive artificial intelligence (AI) detections by imaging modality across gastric anatomical regions. Box plots compare median false‐positive AI detection counts between linked‐color imaging and white‐light imaging across gastric anatomical regions. Boxes represent medians with interquartile ranges. *p < 0.05 indicates statistical significance. Gray boxes represent white‐light imaging, and purple boxes represent linked‐color imaging.

False‐positive AI detections increased with gastric mucosal atrophy severity (Kimura‐Takemoto classification), yet LCI substantially attenuated this effect compared with WLI [15]. In severe atrophy (O‐2/O‐3), LCI reduced median false‐positive detections to 3.5 (Version I) and 2.0 (Version II), compared with markedly higher WLI rates (Figure 5). Similarly, LCI significantly decreased spurious flagging across all gastric anatomical regions versus WLI (*p* < 0.05) (Figure 6).

### 3.5 Qualitative Analysis of False‐Positive AI Detections

Individual false‐positive detection review revealed WLI frequently identified non‐neoplastic structures: mucosal folds (42.9%), bubbles (26.2%), and benign polyps (21.4%). Conversely, LCI's enhanced color contrast facilitated superior AI discrimination of these non‐neoplastic features from genuine lesions, reducing false‐positive detections from mucosal folds (16.7%), bubbles (8.3%), and benign polyps (8.3%). This observation provides a qualitative rationale for LCI's quantitative superiority.

### 3.6 Safety

No adverse events related to the computer‐aided detection device or procedures occurred during this investigation.

## 4 Discussion

This pilot investigation demonstrates that LCI, an image‐enhanced endoscopy modality, substantially enhances AI‐assisted diagnosis by reducing false‐positive detections compared with WLI while maintaining perfect detection of known gastric tumors (6/6 lesions). This finding directly addresses a critical limitation of current AI endoscopy systems: elevated false‐positive detection rates that increase physician fatigue and erode technology confidence [8, 21, 22].

LCI's potential utility in this AI context may stem from its capacity to amplify mucosal and vascular pattern visualization [11, 13, 18]. This type of technology, reported as image‐enhancing mode, accentuates color differences between neoplastic and non‐neoplastic tissue through narrow‐band short‐wavelength light combined with white light [3, 11]. By providing enhanced color contrast, LCI likely supplies AI algorithms with more distinctive analytical features [4, 10, 13]. This enables superior differentiation of genuine lesions from common mimics, including mucosal folds, bubbles, and benign polyps, as our qualitative analysis demonstrates. Such improved discrimination is fundamental for establishing AI as a reliable and efficient clinical partner [23].

While imaging modality comparison was the primary focus, our sub‐analysis of two computer‐aided detection versions provides valuable insight. Version II improved upon Version I in both training case quantity and data diversity [4]. Specifically, Version II received additional training to recognize clearly non‐neoplastic findings such as fundic gland polyps, which frequently generate false‐positive detections in gastric screening [6]. This targeted inclusion likely contributed to marked false‐positive alert reduction, indicating that dataset expansion and qualitative refinement are both essential for improving AI reliability [7, 24]. Version II's enhanced performance demonstrates that algorithmic refinement and targeted training constitute crucial parallel advancement pathways. Importantly, LCI's potential advantage over WLI was observed with both AI versions, suggesting the possible value of this imaging modality when paired with AI [25].

LCI integration with AI represents an emerging diagnostic paradigm. Recent investigations demonstrate that LCI combined with deep learning enhances diagnostic accuracy for various gastric conditions [4, 17, 26]. Our findings extend this literature by showing that LCI not only benefits human endoscopists but also improves AI performance through false‐positive detection reduction. This synergy between advanced imaging and AI technology carries important clinical workflow implications [21].

False‐positive alert reduction improves AI performance metrics while exerting disproportionately positive effects on workflow and human‐machine interaction. Recent gastrointestinal endoscopy studies (e.g., in polyp detection) demonstrate that excessive false‐positive alerts cause “alarm fatigue,” diminishing user confidence and negating intended assistance benefits [7, 23, 26]. This underscores that AI interpretability and data diversity improvements are as vital as the imaging modality itself for sustainable clinical integration [13, 22].

Reducing false positives without compromising sensitivity is desirable to avoid distracting endoscopists and disrupting clinical workflow. Previous studies have highlighted false‐positive alerts as a major limitation of AI‐assisted endoscopy, contributing to alarm fatigue and reduced clinical confidence. Recently, Yasuda et al. reported that image‐enhanced endoscopy modalities can contribute to reducing false‐positive AI detections, supporting the rationale for combining advanced imaging techniques with AI systems to improve clinical usability [4].

These results converge to strongly support the clinical value of an integrated approach. Combining advanced imaging modalities like LCI with continuously improving AI systems represents a powerful strategy [13, 16]. This synergy has the potential to streamline endoscopic workflow, reduce unnecessary biopsies, and ultimately improve patient outcomes in gastric cancer screening.

This study has several limitations. First, its retrospective design and a relatively small sample size. Although the within‐patient design minimizes inter‐patient variability, the imaging sequence was fixed, with WLI observation preceding LCI assessment in all examinations. Therefore, potential sequence‐related bias and second‐observation effects cannot be completely excluded. These effects may include changes in mucosal visibility, endoscopist familiarity with the examined area, or other factors related to the preceding WLI observation. Consequently, the present study cannot determine whether the observed reduction in false‐positive CAD‐EYE detections was attributable solely to LCI itself, nor can it demonstrate the independent superiority of LCI over WLI. Future prospective studies using randomized or counterbalanced imaging sequences are required to clarify the independent effect of LCI on AI‐assisted diagnosis. Accordingly, propensity score matching was applied only as a sensitivity analysis, and the results should be interpreted with this consideration in mind. Another limitation is that pathological confirmation was not available for all AI‐flagged lesions. Although all AI detections were independently reviewed by three expert endoscopists and adjudicated by consensus based on endoscopic appearance and, when available, histologic findings, some lesions classified as false positives were determined based on expert endoscopic assessment alone. Operator‐dependent variations may have influenced results, as the allocation of endoscopists was not randomized. Nevertheless, this pilot provides critical real‐world evidence and a strong rationale for larger, prospective studies validating combined LCI and AI use.

## 5 Conclusion

In this pilot study, LCI assessment performed after WLI observation was associated with significantly fewer false‐positive CAD‐EYE detections without an apparent reduction in sensitivity, and this finding was observed across different CAD‐EYE system versions. However, because the observation sequence was fixed, the independent effect of LCI cannot be determined. Further prospective randomized or counterbalanced studies are required to confirm whether LCI itself improves AI‐assisted gastric cancer diagnosis.

## Author Contributions

Ahmed Nashaat Mohamed contributed to manuscript drafting and revision. Yosuke Minoda conceived and designed the study, performed data analysis, and drafted the manuscript. Ryohei Maruoka, Yusuke Suzuki, Hiroki Fukuya, Hirotaka Tsuru, Masafumi Wada, Yoshimasa Tanaka, Takatoshi Chinen, and Haruei Ogino contributed to data collection and interpretation. Eikichi Ihara, Tomohiko Moriyama, and Yoshihiro Ogawa provided critical revisions and intellectual contributions. All authors approved the final manuscript version.

## Funding

This work received partial support from the National Cancer Center Research and Development Fund (grant number 2023‐A‐15) and JSPS KAKENHI (grant number 23K15044).

## Ethics Statement

**Approval of the research protocol by an Institutional Reviewer Board**: The institutional review board of Kyushu University Hospital approved this study with waived individual informed consent, given the retrospective design (protocol publicly announced via opt‐out mechanism).

## Consent

Waived due to retrospective design.

## Conflicts of Interest

Yosuke Minoda holds research and development consulting contracts with Olympus and MC Medical. Haruei Ogino participates in an endowed course supported by Ono Pharmaceutical, Miyarisan Pharmaceutical, Sanwa Kagaku Kenkyusho, Otsuka Pharmaceutical, Fujifilm Medical, Terumo Corporation, FANCL Corporation, Ohga Pharmacy, and Abbott Japan. Eikichi Ihara has received lecture fees from Takeda Pharmaceutical, Viatris, and EA Pharma, and participated in an endowed course supported by these companies until March 2023. Yoshihiro Ogawa has engaged in collaborative research with Fujifilm Medical and FANCL Corporation. The remaining authors declare no conflicts of interest.

## Supporting information




**FILE S1: TABLE S1A** Covariate balance between CAD version I and CAD version II before and after propensity score matching.
**TABLE S1B** Detailed distribution of gastric mucosal atrophy according to the Kimura–Takemoto Classification.
