# Supplementary material for: Benefit of Linked‐Color Imaging in Artificial Intelligence‐Assisted Diagnosis of Early Gastric Cancer: A Pilot Study With Propensity Score Adjustment
Source: DEN Open. 2026 Jul 28;7(1):e70381. doi: 10.1002/deo2.70381 (PMC13408707; doi:10.1002/deo2.70381)
Supplement: Supplementary file 1 — FILE S1: TABLE S1A Covariate balance between CAD version I and CAD version II before and after propensity score matching. TABLE S1B Detailed distribution of gastric mucosal atrophy according to the Kimura–Takemoto Classification. [file DEO2-7-e70381-s001.docx]

Supplementary Table S1A. Covariate Balance Between CAD Version I and CAD Version II Before and After Propensity Score Matching.

| Variable | CAD Version I before matching (n=39) | CAD Version II before matching (n=24) | SMD before matching | CAD Version I after matching (n=24) | CAD Version II after matching (n=24) | SMD after matching |
| --- | --- | --- | --- | --- | --- | --- |
| Age, median (IQR), years | 63.5 (52.8–73.5) | 62.0 (51.3–73.8) | 0.38 | 62.0 (51.0–72.5) | 62.0 (51.3–73.8) | 0.04 |
| Male sex, n (%) | 18 (46.2) | 14 (58.3) | 0.24 | 14 (58.3) | 14 (58.3) | 0.00 |
| Female sex, n (%) | 21 (53.8) | 10 (41.7) | 0.24 | 10 (41.7) | 10 (41.7) | 0.00 |
| Indication |  |  |  |  |  |  |
| Screening, n (%) | 21 (53.8) | 11 (45.8) | 0.16 | 11 (45.8) | 11 (45.8) | 0.00 |
| Surveillance, n (%) | 18 (46.2) | 11 (45.8) | 0.01 | 13 (54.2) | 11 (45.8) | 0.17 |
| Follow-up after EMR, n (%) | 0 (0) | 2 (8.3) | 0.43 | 0 (0) | 2 (8.3) | 0.43 |
| Gastric mucosal atrophy |  |  |  |  |  |  |
| Closed-type atrophy, n (%) | 30 (76.9) | 20 (83.3) | 0.16 | 20 (83.3) | 20 (83.3) | 0.00 |
| Open-type atrophy, n (%) | 9 (23.1) | 4 (16.7) | 0.16 | 4 (16.7) | 4 (16.7) | 0.00 |

Footnote: Propensity score matching was performed as a supplementary sensitivity analysis for the CAD version-level comparison. The propensity score model included age, sex, clinical indication for endoscopy, and gastric mucosal atrophy status. An absolute SMD <0.1 was considered to indicate adequate balance.

Supplementary Table S1B. Detailed Distribution of Gastric Mucosal Atrophy According to the Kimura–Takemoto Classification.

| Kimura–Takemoto classification | Overall cohort (n=63), n (%) |
| --- | --- |
| C-0 | 31 (49.2) |
| C-1 | 5 (7.9) |
| C-2 | 6 (9.5) |
| C-3 | 8 (12.7) |
| O-1 | 4 (6.3) |
| O-2 | 5 (7.9) |
| O-3 | 4 (6.3) |
| Closed type total (C-0 to C-3) | 50 (79.4) |
| Open type total (O-1 to O-3) | 13 (20.6) |
